# Evaluation of disseminated tumor cells and circulating tumor cells in patients with breast cancer receiving adjuvant zoledronic acid

**DOI:** 10.1038/s41523-021-00323-8

**Published:** 2021-09-06

**Authors:** Neelima Vidula, Sally Greenberg, Laura Petrillo, Jimmy Hwang, Michelle Melisko, Andrei Goga, Mark Moasser, Mark Magbanua, John W. Park, Hope S. Rugo

**Affiliations:** 1 Massachusetts General Hospital Cancer Center, Boston, MA United States; 2 Western Health, Melbourne, Victoria Australia; 3 Massachusetts General Hospital, Division of Palliative Care, Boston, MA United States; 4 University of California San Francisco, UCSF Helen Diller Family Comprehensive Cancer Center Precision Medicine Cancer Building, San Francisco, CA United States

**Keywords:** Metastasis, Breast cancer

## Abstract

We evaluated disseminated tumor cells (DTCs) and circulating tumor cells (CTCs) in patients with stage I-III breast cancer with >4 MM/mL DTC at baseline who received adjuvant zoledronic acid (ZOL). ZOL was administered every 4 weeks for 24 months, and patients underwent bone marrow aspiration at baseline, and 12 and 24 months of ZOL. Complete DTC response (<4 DTC/mL), serial CTCs, survival, recurrence, and toxicity were determined. Forty-five patients received ZOL. Median baseline DTC was 13.3/mL. Significant reduction in median DTC occurred from baseline to 12 months, and 24 months. Complete DTC response was seen in 32% at 12 months, and 26% at 24 months. Nine patients developed recurrence. Baseline DTC > 30/mL and CTC > 0.8/mL were significantly associated with recurrence and death. Serial reduction in DTCs occurred. Higher baseline DTC > 30/mL and CTC > 0.8/mL correlated with recurrence and death.

## Introduction

Breast cancer is the most common cancer affecting women^1^. The development of distant metastases is the predominant cause of breast cancer-related mortality. Detection of circulating tumor cells (CTC) in the blood and disseminated tumor cells (DTC) in the bone marrow may provide early evidence of risk for subsequent metastatic disease. The bone marrow may act as a sanctuary for DTCs due to the abundance of growth factors^2^ and by acting as a relative barrier to systemic adjuvant chemotherapy^3^. Although there is evidence that DTCs and CTCs have prognostic significance^3–7^, the optimal management of patients with these findings remains unclear^8^.

Bisphosphonates inhibit osteoclast-mediated bone resorption and are effective in the prevention and treatment of osteoporosis^9^, and in the reduction of skeletal complications in metastatic cancer^10^. Zoledronic acid (ZOL) is a highly potent, heterocyclic nitrogen-containing third-generation bisphosphonate, which is effective in the treatment of bone metastases in patients with breast cancer^11^. In addition to preventing bone-related complications, bisphosphonates may also play a role in reducing the risk of breast cancer recurrence^12,13^. The potential mechanisms for antitumor effects of bisphosphonates are still under investigation but in vitro and preclinical studies have shown that bisphosphonates reduce the ability of tumor cells to adhere to bone, invade, proliferate and stimulate apoptosis, and impact tumor migration and invasion^14^.

We developed an immunomagnetic enrichment with a flow cytometry system for detection of DTCs using the EPCAM antibody^15,16^. We designed a prospective pilot study to evaluate serial DTCs and CTCs in patients with stage I-III breast cancer with DTCs present after completion of neoadjuvant or adjuvant chemotherapy (as indicated) during treatment with adjuvant ZOL every 4 weeks for 24 months. The primary endpoint was to determine the proportion of patients who achieved a complete DTC response after treatment with ZOL. Exploratory endpoints included assessing overall survival, recurrence, and toxicity, as well as serial evaluation of CTCs.

## Results

### Patient demographics

Forty-five patients with evidence of DTCs and clinical stage I, II, or III breast cancer were enrolled between October 2004 and October 2007 at the University of California San Francisco Comprehensive Cancer Center. Baseline demographics and disease characteristics are displayed in Table 1. More patients (58%, *n* = 26) were premenopausal and 91% (41) had stage II or III disease. Ninety-six percent (43) of patients received chemotherapy. The two patients who did not receive chemotherapy had hormone receptor (HR) positive tumors. Fifty-six percent (25) of patients had hormone receptor (HR) positive disease and 18% (8) had human epidermal growth factor 2 (HER2) positive tumors. All patients with HR-positive disease were treated with adjuvant hormonal therapy (tamoxifen or aromatase inhibitor), and all patients with HER2 positive disease had received trastuzumab.

**Table 1:** Baseline patient characteristics.

Characteristic	Patients (*n* = 45)
Mean age (years)	46 (range: 29–64)
Premenopausal	26 (58%)
Stage of breast cancer*	
I	4 (9%)
II	26 (58%)
III	15 (33%)
Estrogen Receptor Positive	25 (56%)
Progesterone Receptor Positive	21 (47%)
HER2 positive	8 (18%)
Chemotherapy (adjuvant and neoadjuvant)	43 (96%)
Neoadjuvant chemotherapy	20 (44%)
Hormonal therapy	25 (56%)

^*^Stage according to the *American Joint Committee on Cancer Staging Manual*, 6th ed. Clinical stage was used for patients who received neoadjuvant chemotherapy and pathological stage was used for those patients who received adjuvant or no chemotherapy.

In this study with more than 10 years of follow-up, four patients withdrew for reasons other than progressive disease (one due to toxicity of intravenous site irritation, one for noncompliance, and two for personal reasons).

### DTC analyses

Of the 45 patients enrolled, DTC analysis was available for 82% (37) at 12 months and 76% (34) at 24 months. Among 34 patients at 0–24 months, one patient did not have DTC results at 24 months due to an inadequate bone marrow aspiration, and therefore was not included in the 24-month analyses done. Thirty-three patients had DTC results available for the serial analyses in the study, as shown in Table 2. The median DTC at baseline for all patients enrolled was 13.3 cells/mL (range 4–332.9/mL). Baseline DTC level was not significantly correlated with tumor HR or HER2 expression. There was a significant reduction in DTCs from baseline to 12 months with a median reduction of 6.5 cells/mL (range −57.2 to 23.6, *p* < 0.001) and from baseline to 24 months with a median reduction of 4.6 cells/mL (range −43.1 to 160.1/mL, *p* < 0.001) by signed rank test (Table 2). For the 33 patients who had bone marrow aspirates at all three time points, there was a reduction in DTCs over time: baseline median 11.5 cells/mL (range 4.0–59.3/mL), 12 months median 6.8 cells/mL (range 0–28.5/mL), and 24-month median 6.5 cells/mL (range 0.3–168.6/mL).

**Table 2:** Change in DTCs over time.

	Number	Median (range) DTC/mL	Recurrence ate [*n* (%)]
All available results			
Baseline	45	13.3 (4–332.9)	
Reduction from 0 to 12 months	37	−6.5 (−57.2–23.6)	3 (33.3%)
Reduction from 0 to 24 months	34	−4.55 (−43.1–160.1)	5 (55.6%)
Results for patients for whom BMA available at all three time points			
Baseline	33	11.5 (4.0–59.3)	
12 months	33	6.8 (0–28.5)	3 (33.3%)
24 months	33	6.5 (0.3–168.6)	5 (55.6%)

*DTC* Disseminated tumor cell, *BMA* Bone marrow aspirate.

A significant number of participants cleared their marrow of DTCs during the study period (defined as <4 DTC/mL): 32% (12) at 12 months and 26% at 24 months. There was no association between the proportion of patients that cleared their DTCs based on HR status at 12 months (31 vs. 37%) or at 24 months (32 vs. 33%).

### CTC analyses

Median CTC at baseline was 0.2/mL (range 0–8.6) for all 45 participants. There was no significant change in CTCs over time (Table 3), although only 51% (23) had results at 24 months.

**Table 3:** Change in CTCs over time.

	Number	Median (range) CTC/mL	Recurrence Rate [*n* (%)]
Baseline	45	0.2 (0– 8.6)	
6 months	30	0.25 (0–1.2)	1 (11.1%)
12 months	22	0.0 (0.0–1.5)	3 (33.3%)
18 months	17	0.1 (0.0–5.6)	4 (44.4%)
24 months	23	0.2 (0–12)	5 (55.6%)

*CTC* circulating tumor cell.

### Recurrence

The median follow-up was 123 months (range 5–154 months). We evaluated the development of local or distant recurrence. Nine patients (20%) developed disease recurrence (seven distant recurrences and two local recurrences). The median time from diagnosis to recurrence was 21 months (range 6–105). The disease and treatment characteristics of patients who developed recurrence are shown in Table 4. There was no association of recurrence with HR or HER2 status, tumor size, and stage at diagnosis. Of those with recurrence, 67 percent of patients (six) had triple-negative disease, 22% (two) patients had estrogen receptor (ER) positive/HER2+ disease and the remaining patient had HR+/HER2- disease. Thirty-three percent (three) had central nervous system metastases as the first site of recurrence, 22% had a recurrence in both the bone and viscera (two), 11% had only visceral involvement (one), and 22% had local recurrence (two). One patient (11%) had a malignant pleural effusion as a first site of relapse.

**Table 4:** Characteristics of Patients who Developed Metastatic/Recurrent Disease.

Stage	Grade	ER/PR/HER2 status	Systemic therapy (prior to ZOL)	Baseline DTC/mL	M12 DTC/mL	M24 DTC/mL	Baseline CTC/mL	M6 CTC/mL	M12 CTC/mL	M18 CTC/mL	M24 CTC/mL	Time to recurrence from diagnosis (months)	Site of recurrence	Time to Overall Survival (months)
III	2	TNBC	AC/T	30.9	–	–	8.6	–	–	–	–	5.5	CNS	8.1
II	2	TNBC	AC/T	332.9	–	–	0	–	–	–	–	11.5	CNS	24.8
III	2	TNBC	AC/T	30.1	–		0.9	0.2	–	–	–	12	Visceral + skeletal	21.8
I	3	TNBC	AC/T	14.2	–	–	2.4	–	–	–	–	17.5	Visceral + skeletal	34.5
II	3	TNBC	AC/T	32	–	–	0	0	–	–	–	21	CNS	22.9
III	2	ER + /PR-/HER2 +	AC/T + exemestane + trastuzumab	8.5	6.9	168.6	0.5	0.6			0.3	39	Visceral	71.4
III	3	TNBC	AC/T	13.3	–		0.1	–		1	–	43	Local recurrence	Alive
II	3	ER + /PR + /HER2 +	AC/T + trastuzumab	5	0	3.3	0.2	0	0	–	0.1	79	Local recurrence	Alive
III	2	ER + /PR + /HER2-	AC/T	22.5	3.1	–	0	0.1	–	–	–	111	Pleural effusion	Alive

AC/T: 4 cycles of doxorubicin (A) and cyclophosphamide (C) followed by 4 cycles of paclitaxel (T).

*ER e*strogen receptor, *PR* progesterone receptor,

*HER2* human epidermal growth factor receptor 2,

*OS* months from diagnosis to death.

*M* month.

Using the recurrence rate and the Fisher’s Exact test, baseline DTCs >30/mL was associated with a higher risk of recurrence. Fifty percent of patients (four of eight) with baseline DTC greater than 30/mL developed recurrence whereas only 13.5% (five of 37) of patients with baseline DTC less than 30/mL experienced recurrence (odds-ratio = 6.4, *p* = 0.039). All three patients who had central nervous system recurrence had baseline DTC greater than 30/mL (*p* < 0.001). Of the patients who developed recurrence, four patients had ≥1 serial bone marrow aspiration. Two of four (50%) had increasing DTC detected before recurrence. In contrast, of the 32 disease-free patients who had bone marrow aspirations available at all three-time points, 72% (23) had a decrease in bone marrow DTC from baseline to 24 months. During this study, six patients died (13.3%). Of these six patients, 67% (four) had a baseline DTC > 30/mL, which was associated with a higher risk of death (50% > 30 vs. 5% < 30, odds-ratio = 17.5, *p* = 0.006).

A multivariable regression analysis was performed to determine whether DTCs are independently associated with recurrence. However, there were not enough patients with recurrence (*n* = 9) and many factors were linearly related, and consequently this was not significant.

A higher CTC level (>0.8/mL) at baseline was also found to be associated with a higher risk of recurrence. Sixty percent of patients (three of five) with CTC > 0.8/mL experienced recurrence compared with 15% of patients (six of 40) with CTC < 0.8/mL (odds-ratio = 8.5, *p* = 0.047). In addition, baseline CTC > 0.8/mL was found to be associated with a higher risk of death (60% > 0.8/mL vs. 8% < 0.8/mL, odds-ratio = 18.5, *p* = 0.013).

### Urinary n-telopeptide

**Fig. 1:**
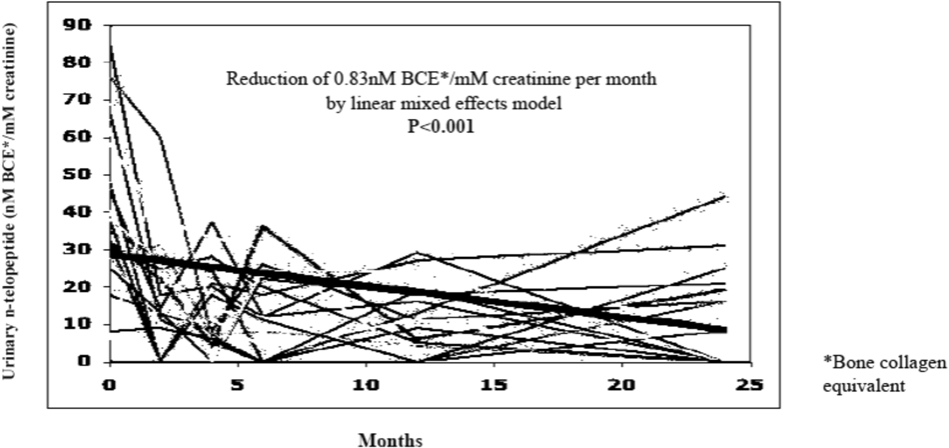
Change in urinary n-telopeptide in 34 evaluable patients with baseline urinary n-telopeptide results. A reduction of 0.83 nM bone collagen equivalent (BCE) per mM creatinine per month (*p* < 0.001) was seen over the 24-month study period.

Thirty-four evaluable patients had baseline urinary n-telopeptide results. A reduction of 0.83 nM bone collagen equivalent (BCE) per mM creatinine per month (*p* < 0.001) was seen over the 24-month study period (Fig. 1), consistent with a treatment effect of ZOL.

### Toxicity

Toxicity data were available for all 45 patients enrolled. ZOL was well tolerated. Seventy-three percent of patients (33 patients) reported grade 1 or 2 toxicity. The most commonly reported toxicities, occurring in more than 5% of patients, included joint pain and muscle aches in 56% (25 patients), fatigue in 27% (12), fever in 9% (four), and nausea in 7% (three). One patient withdrew from the study due to recurrent skin irritation at the intravenous site, and one patient required a dose delay for a transient elevation in creatinine. There were no occurrences of jaw osteonecrosis.

Data from this study are provided in this manuscript and also available upon request.

## Discussion

In this study, we treated patients with stage I-III breast cancer with evidence of DTCs following completion of neoadjuvant or adjuvant chemotherapy and HER2 targeted therapy (if indicated) with adjuvant ZOL for 24 months and evaluated serial quantitative changes in DTCs and CTCs. We did not note any significant association between the DTCs and tumor subtype, suggesting that DTCs are biomarkers that are independent of receptor subtype. This study is limited by its sample size of 45 patients and heterogeneous patient population with varying disease subtypes and menopausal status. Furthermore, patients included in this study received heterogeneous treatment appropriate to their subtype of breast cancer, and the time at which baseline DTCs were collected varied, as elaborated in the methodology. Additionally, given the pilot nature of this study, a control arm of patients not receiving ZOL was not included, so the impact of time or assay dependent changes in DTC measurements is not known.

A significant reduction in median DTC from baseline to 12 months and from baseline to 24 months was observed in this study of patients treated with ZOL. Furthermore, a significant quantitative reduction in DTC was observed in 33 patients who had bone marrow assessments available at baseline, 12, and 24 months. Additionally, 32% of patients in the study cleared DTCs at 12 months, regardless of HR status, and 26% of patients cleared DTCs at 24 months, meeting the primary endpoint of this study. However, causality cannot be attributed to ZOL given the nonrandomized nature of this study, and the impact of time alone on DTC clearance is not known. A recent randomized study demonstrated that the addition of ZOL to standard adjuvant chemotherapy and/or hormone therapy increased rates of DTC clearance compared to standard adjuvant therapy alone^17^. It is not known whether ZOL may clear some DTCs but not others, and whether this may be a biological difference of the DTCs.

A number of studies have evaluated the impact of bisphosphonates on breast cancer outcomes, and some have demonstrated a reduction in bone metastases^12,18,19^, improved disease-free survival^18,20–22^, and/or improved overall survival^19^. Our work supports the results of a large meta-analysis that demonstrated a significant decrease in recurrence, including recurrence at distant sites and in the bone, and mortality from breast cancer with the use of bisphosphonates among 11,767 postmenopausal women^19^. An improvement in outcomes may potentially be mediated in part by a reduction of DTCs with bisphosphonates, as demonstrated by a study noting better outcomes and increased DTC clearance with the addition of zoledronic acid to adjuvant chemotherapy^23^, and a second study noting increased clearance of DTCs with the addition of zoledronic acid to neoadjuvant chemotherapy^24^.

The risk of recurrence and death in our study was significantly higher for patients with more than 30 DTC/mL at baseline. The detection of DTCs in most published studies has been via immunohistochemical stains using monoclonal antibodies to cytokeratins or epithelial cell membranes, resulting in a qualitative assessment as to the presence or absence of these cells^25–27^. In contrast, our study utilized IF/FC to provide a quantitative result^15,28^. These prior qualitative studies have established an association between the presence of DTC and poor outcomes^4^. Our study suggests that a higher DTC count results in a more substantial risk of recurrence than the presence of these cells alone, as we observed a higher risk of recurrence in patients with DTCs >30/mL, within our population of exclusively DTC positive patients. Due to the small number of events in our trial, we were not able to assess the impact of DTCs on bone-specific recurrence but this remains an intriguing direction for future research. We also could not assess the prognostic impact of a bisphosphonate in DTC positive patients, given the nonrandomized nature of our study but another recent study has demonstrated that treatment with a bisphosphonate was an independent prognostic factor in patients positive for DTCs^29^. Furthermore, the mechanism by which ZOL may have an impact on recurrence is being studied. Animal models demonstrate that ZOL directly affects the tumor growth and tumor burden of bone and visceral metastases, possibly by impacting tumor migration and invasion^14,30,31^. It is intriguing that some patients in this study developed CNS recurrence. Understanding the association between DTCs and CNS metastases is a potential area for future research.

As an exploratory endpoint, we evaluated changes in CTCs with treatment. We observed that a greater CTC count (>0.8/mL) at baseline was associated with a higher risk of recurrence and death, similar to our findings that a higher DTC count correlated with risk of recurrence and death. Other studies have demonstrated that the persistence of CTCs after neoadjuvant therapy is a prognostic factor^32^ and that an increasing CTC count correlates with worse survival outcomes, similar to our findings. A recent study of patients with HR + /HER2- stage II or III breast cancer demonstrated that the presence of CTCs five years after diagnosis was associated with an increased risk of late recurrence^5,7^. In addition, studies have demonstrated that circulating tumor DNA (ctDNA), another peripheral biomarker, present after neoadjuvant therapy is a prognostic factor^33^, and ctDNA present after adjuvant treatment predicts relapse^34,35^. Taken together, these data suggest that CTCs and ctDNA are promising biomarkers for breast cancer recurrence, and a study has also demonstrated that these peripheral assays may provide complementary information for the prediction of relapse, disease-free survival, and overall survival^33^. Further research is needed to determine how DTCs, CTCs, and ctDNA relate to one another, as they may represent discrete mechanisms of tumor cell metastasis^33,36^, and a better understanding of these biomarkers may be helpful to identify those treatment settings in which each of these markers has the most utility. CTCs and ctDNA appear to be a promising approach that could avoid the technical challenges and burden to patients associated with the bone marrow biopsy required for the quantification of DTCs.

In conclusion, in this pilot study of patients with stage I-III breast cancer who received adjuvant ZOL, we observed a reduction in serial DTCs. Higher baseline DTC and CTC were found to be predictive of recurrence and death.

## Methods

### Patients

Patients with stage I-III invasive breast cancer underwent unilateral bone marrow aspiration to screen for the presence of DTCs (Clinical Trials.gov Identifier: NCT00295867, trial registered November 3, 2004). Written informed consent was obtained from all patients. The study was conducted in accordance with the Declaration of Helsinki and Good Clinical Practice Guidelines. The institutional review board for The University of California San Francisco approved the study. Women over the age of 18 years with stage I-III breast cancer (confirmed histologically or cytologically) who had >4 DTC/mL in the bone marrow were eligible. Baseline bone marrow aspiration occurred at the time of surgery or after surgery in patients who received neoadjuvant chemotherapy or hormone therapy, at least 3 weeks after completion of adjuvant chemotherapy in patients who received adjuvant chemotherapy (including completion of adjuvant trastuzumab if indicated), or at the time of definitive surgery in patients who were to receive hormonal therapy alone. In patients who had bone marrow aspiration after surgery, this was obtained within 1 year after surgery or completion of adjuvant therapy. However, the majority of bone marrow aspirations were obtained at the time of surgery. Patients could receive concomitant hormonal therapy and/or radiation (if indicated; completion of these was not required prior to bone marrow aspiration and study participation). Cytotoxic anticancer therapy was not allowed in the study. Patients were required to have an adequate renal function (creatinine ≤ upper limit of normal) and normal liver function (total bilirubin, alkaline phosphatase, and aspartate aminotransferase). Exclusion criteria included history of allergy to bisphosphonates, renal insufficiency (serum creatinine > upper limit of normal or a creatinine clearance <50 mL/min), Karnofsky Performance Status <90%, pregnancy, and any significant comorbid medical condition that could interfere with treatment. The use of any other bone directed therapy during the study period was not allowed.

### Treatment

ZOL was administered intravenously, over 15 min, monthly for 24 months at a dose of 4 mg (provided by Novartis Oncology). A reduced dose was administered if a reduction in creatinine clearance (CrCl) using the Cockcroft-Gault formula occurred, based on the package insert^37^. Patients were instructed to take a multivitamin containing 400 IU of vitamin D as well as an oral calcium supplement of at least 500 mg daily.

### DTC and CTC Detection/Quantification

Screening bone marrow aspiration from the posterior iliac crest was performed to evaluate for DTCs. The timing of baseline bone marrow aspiration is as described above. Patients underwent repeat bone marrow aspirations and DTCs were measured at 12 and 24 months (end of therapy) following treatment initiation. Peripheral blood was drawn for CTCs at screening and 6, 12, 18, and 24 months after starting treatment.

DTCs and CTCs were enumerated using IE/FC (immunomagnetic enrichment and flow cytometry) as previously described^15,16^, which is a system we developed. Briefly, magnetic beads coated with a monoclonal antibody to EPCAM were added to whole blood to enrich for EPCAM-expressing cells and the sample was subjected to flow cytometry to detect CTCs or DTCs, defined as nucleated cells that are EPCAM-positive and CD45 negative. A positive test for DTCs was defined as more than four DTC per mL, which is greater than 2.5 standard deviations above the mean value in normal bone marrow samples with this quantification system (as studied in 50 normal marrow samples by Park et al.^38^ and validated in subsequent studies from this laboratory^16^). Additional work has demonstrated that DTCs detected by this method have prognostic significance, contributing to an adverse outcome in early-stage breast cancer^16^. A study has also confirmed that DTCs may have characteristics similar to luminal or basal breast cancer, as well as express oncogenes, confirming their status as the tumor cells^36^.

A positive test for CTCs was defined as more than 0.8 CTC per mL. We used a concentration based unit (CTC/ml) because the amount of blood drawn was not precisely fixed in the protocol. For example, the well-known CellSearch assay defines positive as five CTC per 7.5 mL CellSave^TM^ tube, since all samples must be submitted using these specific collection tubes; this is therefore 0.67 CTC per mL. For analysis, we evaluated several values of DTC and CTC to identify a threshold predictive of recurrence or death.

### Additional assessments

Serum creatinine was measured monthly prior to each ZOL infusion. Patients underwent history and physical examination at study screening and every 4 months during the 24-month study period. Toxicity, recurrence and survival were assessed at each visit and every 6 months after completion of study therapy.

Urinary n-telopeptide was collected at baseline and 2, 4, 6, 12, and 24 months after study enrollment, as well as 6 months after the end of the study. Bisphosphonate use has been shown to decrease urinary excretion of n-telopeptide, a marker of bone turnover^39^.

### Statistical analysis

The primary endpoint was achieving a complete DTC response, defined as reduction of DTCs to less than 4 DTC/mL. Treatment was deemed successful if more than five patients had <4 DTC/mL at the completion of 12 or 24 months of ZOL therapy, based on the null hypothesis that 5% or fewer of all patients with initial bone marrow DTC > 4 DTC/mL would have subsequent bone marrow DTC ≤ 4 DTC/mL if untreated. The alternate hypothesis was assumed to be a success rate of 22% or greater of patients with DTC ≤ 4 DTC/mL in the treated population. A sample size of 35 or more patients was required to have 90% power to determine the significance of the hypothesis based on a binomial distribution with a type I error of 5%. At the time of study design, data on serial measurements from patients with initial bone marrow DTC > 4 DTC/mL was not available, so the null hypothesis described above was used, which is reasonable as another group has since demonstrated that DTCs are detectable in patients with breast cancer 3 years after initial diagnosis and management^40^. For evaluation of the primary endpoint, patients were included if they received at least six months of ZOL. Additional exploratory endpoints included reduction in DTCs and CTCs over time, recurrence (local or distant), overall survival and toxicity. The recurrence rate and the Fisher’s Exact test were used to assess the impact of baseline parameters of DTCs and CTCs on the risk of recurrence and death. The linear mixed-effects model was used to determine the change in urinary n-telopeptide over time.

### Reporting Summary

Further information on research design is available in the Nature Research Reporting Summary linked to this article.

## Supplementary information

Supplementary Information

Reporting Summary

## Data Availability

The datasets generated during and analyzed in the current study are available from the corresponding author on reasonable request and with permission of the IRB. These are not publically available as they contain patient information. Please also note that Supplemental Table 1 includes data included in this paper.
