# Supplementary material for: Evaluation of disseminated tumor cells and circulating tumor cells in patients with breast cancer receiving adjuvant zoledronic acid
Source: NPJ Breast Cancer. 2021 Sep 6;7:113. doi: 10.1038/s41523-021-00323-8 (PMC8421499; doi:10.1038/s41523-021-00323-8)
Supplement: Supplementary file 1 — Supplementary Information [file 41523_2021_323_MOESM1_ESM.pdf]

Supplemental Table 1. Patient characteristics. BS=baseline. DD=standard dose dense chemotherapy

Study ID	BMA_BS (DTC/mL)	BMA_M12 (DTC/mL)	BMA_M24 (DTC/mL)	PB_BS (CTC/mL)	PB_M6 (CTC/mL)	PB_M12 (CTC/mL)	PB_M18 (CTC/mL)	PB_M24 (CTC/mL)	Chemo	Cycles	Hormone Therapy	Stage	ER-Pos	PR-Pos	HER2 neu Pos	TNBC	Recurrence	Brain_mets	Visceral_mets	Bone_mets	Local_recurrence	Death
E01	13	10.3	5.6	0.3	0		0	0	FEC	6 cycles	Arimidex	IIA	Y	Y	N	N	N	N	N	N	N	N
E02	8.3	10	4.2	0.3	0	0			AC/Taxotere	4 cycles, 3 cycles	0	IIB	N	N	N	Y	N	N	N	N	N	N
E03	11.5	12.3	17.4	0.5	0	0	0.5	0	Epirubicin&cytoxan/Taxol /Xeloda	4 cycles, 10 cycle, 8 cycles	0	IIA	N	N	N	Y	N	N	N	N	N	N
E04	6.1	8.4	10.2	0	0.6	0.3	5.6	0.3	AC/Taxotere	4 cycles, 4 cycles	0	IIIA	N	N	N	Y	N	N	N	N	N	N
E05	11.5	5	6.1	0	0.2	0.1	0.1		AC	4 cycles	Tamoxifen	I	Y	Y	N	N	N	N	N	N	N	N
E06	23.8	17.2		0	1.2				AC/Taxol/H	8 cycles	Tamoxifen	IIB	Y	N	Y	N	N	N	N	N	N	N
E07	19.7	6.4	9.9	0	0.4	0		0.1	AC/Taxol	8 cycles	0	IIA	N	N	N	Y	N	N	N	N	N	N
E08	10.5	1.9	0.3	0.3	0.4			0.1	AC/Taxol	8 cycles	0	IIB	N	N	N	Y	N	N	N	N	N	N
E09	4.9	28.5	4.9	0.1	0.2	0.2		0.2	AC/Taxol	DD	0	IIIA	N	N	N	Y	N	N	N	N	N	N
E10	9.4	9.4	14.5	0	0			0.1	AC/Taxol	DD	Arimidex	IIIA	Y	Y	N	N	N	N	N	N	N	N
E11	22.9	17.1	13.4	0	0	1.5		0	AC/Taxol	DD	Tamoxifen	IIIC	Y	Y	N	N	N	N	N	N	N	N
E12	32			0	0				AC/Taxol	DD	0	IIB	N	N	N	Y	Y	Y	N	N	N	Y
E13	14.2			2.4					AC/Taxol	DD	0	II	N	N	N	Y	Y	N	Y	Y	N	Y
E14	8.5	6.9	168.6	0.5	0.6			0.3	FEC/Taxol	6 cycles	lupron/Zoladex/Arimidex/Tamoxifen interrupted/Aromasin	IIIA	Y	N	Y	N	Y	N	Y	N	N	Y
E15	6.3	6.1	2.5	0.3		0			AC/Taxol	8 cycles	0	II	N	N	Y	N	N	N	N	N	N	N
E16	6.1			1	0.8				AC/Taxol	8 cycles	Arimidex	II	Y	Y	N	N	N	N	N	N	N	N
E17	13.3		5.8	0.1			1		AC/Taxol	8 cycles	0	IIIC	N	N	N	Y	Y	N	N	N	Y	N
E18	22.5	3.1		0	0.1				AC/Taxol	8 cycles		IIIC	Y	Y	N	N	Y	N	N	N	N	N
E19	17.1			0.6					AC/Abraxane	4 cycles	Oophorectomy	IIA	Y	Y	N	N	N	N	N	N	N	N
E20	6.4	6.8	7.7	0.2			0.1		AC/Taxol/Taxotere	3 cycles, 1 cycle, 3 cycles	Arimidex/aromasin/Zoladex/Femara	IIB	Y	Y	N	N	N	N	N	N	N	N
E21	40.3	8.3	6.5	0.5	0.1		0.1		AC/Taxol	8 cycles	Femara	IIIC	Y	Y	N	N	N	N	N	N	N	N
E22	59.3	2.1	25.1	0.2	0.3		0		AC/Taxol	DD	0	IIB	N	N	N	Y	N	N	N	N	N	N
E23	30.9			8.6					AC/Taxol	8 cycles	0	IIIC	N	N	N	Y	Y	Y	N	N	N	Y
E24	20.8	6.5		0		0		0	AC/Taxol	8 cycles	Arimidex/Tamoxifen	I	Y	Y	N	N	N	N	N	N	N	N
E25	6.5	1.9	2.5	0.3	0			0	AC/Taxol	8 cycles	Arimidex/Femara	IIIA	Y	N	N	N	N	N	N	N	N	N
E26	30.1			0.9	0.2				AC/Taxol	8 cycles	0	IIIA	N	N	N	Y	Y	N	Y	Y	N	Y
E27	28.9	12.9	4.5	0.4					AC/Taxotere	8 cycles	Arimidex/Tamoxifen	IIB	Y	Y	N	N	N	N	N	N	N	N
E28	5.7	4.2	2.2	0	0	0.6		1	AC/Taxol	8 cycles	Arimidex	IIA	Y	N	N	N	N	N	N	N	N	N
E29	332.9			0					AC/Taxol	8 cycles	0	IIB	N	N	N	Y	Y	Y	N	N	N	Y
E30	4.0	17.3	4	0	0.3			0.7	AC	4 cycles	0	I	N	N	N	Y	N	N	N	N	N	N
E31	45.5	1	2.9	0.4	0.3		0.1		AC/Taxol	8 cycles	0	IIA	N	N	N	Y	N	N	N	N	N	N
E32	5.0	0	3.3	0.2	0	0		0.1	AC/Taxol/H	8 cycles		II	Y	Y	Y	N	Y	N	N	N	Y	N
E33	24.3	12.6	11.3	0		0	0.3	0.1	AC/Taxol	8 cycles	Zoladex/Arimidex	IIIA	Y	Y	N	N	N	N	N	N	N	N
E34	10.8	3.4	15.4	0		0	0.1	0.4	AC/Taxol	4 cycles	0	IIB	N	N	N	Y	N	N	N	N	N	N
E35	10.5	1.1	0.7	0.3	0.9	0.7	0	0.3	AC/Taxol	4 cycles	Arimidex	IIB	Y	Y	N	N	N	N	N	N	N	N
E36	26.2	17.7	11	0.1	0.3	1.4			AC/Taxol/Herceptin	4 cycles	Arimidex/Zoladex	IIIA	Y	Y	Y	N	N	N	N	N	N	N
E37	21.1	7.8	4.2	0		0.3	0.3	0.8	AC/Taxol	4 cycles	0	IIB	N	N	N	Y	N	N	N	N	N	N
E38	12.8	0	9.4	0.1		0.1	0.1	0.4	AC/Taxotere	4 cycles	0	IIB	N	N	N	Y	N	N	N	N	N	N
E39	50.9	1.1	7.8	0		0	0	0.3	AC/Taxol	4 cycles	Tamoxifen	IIA	Y	Y	N	N	N	N	N	N	N	N
E40	13.6	10.8	8.6	0.2	0			0.4	0	0	Zoladex/Arimidex	IIA	Y	Y	N	N	N	N	N	N	N	N
E41	5.6	3.1	6.7	0	0.8				AC/Taxol/Herceptin	3 cycles		IIA	Y	Y	Y	N	N	N	N	N	N	N
E42	10.5	9.8	4.6	0.4		0.1		0	0	0	Tamoxifen	I	Y	Y	N	N	N	N	N	N	N	N
E43	22.1	11.3	21.7	0.4	0.9	0			AC/Taxol	4 cycles	Tamoxifen	IIIA	Y	Y	N	N	N	N	N	N	N	N
E44	6	2.2	2.7	1.5	0.7	0	1.6		AC/Taxol/Herceptin	8 cycles	0	IIIA	N	N	Y	N	N	N	N	N	N	N
E45	18.1	5.9	20.7	0.7	0.4	0	0.4	12	AC/Taxol	4 cycles	Tamoxifen		Y	Y	Y	N	N	N	N	N	N	N
