# Supplementary material for: Evaluation of disseminated tumor cells and circulating tumor cells in patients with breast cancer receiving adjuvant zoledronic acid
Source: NPJ Breast Cancer. 2021 Sep 6;7:113. doi: 10.1038/s41523-021-00323-8 (PMC8421499; doi:10.1038/s41523-021-00323-8)
Supplement: Supplementary file 2 — Reporting Summary [file 41523_2021_323_MOESM2_ESM.pdf]

nature research

Corresponding author(s): Neelima Vidula

Last updated by author(s): Dec 28, 2020

Reporting Summary

Nature Research wishes to improve the reproducibility of the work that we publish. This form provides structure for consistency and transparency in reporting. For further information on Nature Research policies, see our Editorial Policies and the Editorial Policy Checklist.

Statistics

For all statistical analyses, confirm that the following items are present in the figure legend, table legend, main text, or Methods section.

n/a	Confirmed	
☐	☒	The exact sample size (n) for each experimental group/condition, given as a discrete number and unit of measurement
☐	☒	A statement on whether measurements were taken from distinct samples or whether the same sample was measured repeatedly
☐	☒	The statistical test(s) used AND whether they are one- or two-sided<br>*Only common tests should be described solely by name; describe more complex techniques in the Methods section.*
☒	☐	A description of all covariates tested
☒	☐	A description of any assumptions or corrections, such as tests of normality and adjustment for multiple comparisons
☒	☐	A full description of the statistical parameters including central tendency (e.g. means) or other basic estimates (e.g. regression coefficient) AND variation (e.g. standard deviation) or associated estimates of uncertainty (e.g. confidence intervals)
☐	☒	For null hypothesis testing, the test statistic (e.g. F, t, r) with confidence intervals, effect sizes, degrees of freedom and P value noted<br>*Give P values as exact values whenever suitable.*
☒	☐	For Bayesian analysis, information on the choice of priors and Markov chain Monte Carlo settings
☒	☐	For hierarchical and complex designs, identification of the appropriate level for tests and full reporting of outcomes
☒	☐	Estimates of effect sizes (e.g. Cohen's d, Pearson's r), indicating how they were calculated

Our web collection on statistics for biologists contains articles on many of the points above.

Software and code

Policy information about availability of computer code

Data collection	No software was used
Data analysis	Analysis performed by study statistician.

For manuscripts utilizing custom algorithms or software that are central to the research but not yet described in published literature, software must be made available to editors and reviewers. We strongly encourage code deposition in a community repository (e.g. GitHub). See the Nature Research guidelines for submitting code & software for further information.

Data

Policy information about availability of data

All manuscripts must include a data availability statement. This statement should provide the following information, where applicable:

- Accession codes, unique identifiers, or web links for publicly available datasets
- A list of figures that have associated raw data
- A description of any restrictions on data availability

The datasets generated during and analyzed during the current study are available from the corresponding author on reasonable request and with permission of the IRB. These are not publically available as they contain patient information.

Field-specific reporting

Please select the one below that is the best fit for your research. If you are not sure, read the appropriate sections before making your selection.

- [x] Life sciences
- [ ] Behavioural & social sciences
- [ ] Ecological, evolutionary & environmental sciences

For a reference copy of the document with all sections, see nature.com/documents/nr-reporting-summary-flat.pdf

Life sciences study design

All studies must disclose on these points even when the disclosure is negative.

Sample size	The primary endpoint was achieving a complete DTC response, defined as reduction of DTCs to less than 4MM/mL. Treatment was deemed successful if more than 5 patients had <4 MM/mL at the completion of 12 or 24 months of ZOL therapy, based on the null hypothesis that 5% or fewer of all patients with initial bone marrow DTC > 4 MM/mL would have subsequent bone marrow DTC ≤ 4 MM/mL if untreated. The alternate hypothesis was assumed to be a success rate of 22% or greater of patients with DTC ≤ 4 MM/mL in the treated population. A sample size of 35 or more patients was required to have 90% power to determine the significance of the hypothesis based on a binomial distribution with a type I error of 5%. At the time of study design, data on serial measurements from patients with initial bone marrow DTC > 4 MM/mL was not available, so the null hypothesis described above was used, which is reasonable as another group has since demonstrated that DTCs are detectable in patients with breast cancer 3 years after initial diagnosis and management 40.
Data exclusions	None
Replication	This was a clinical trial and conducted in accordance with standard guidelines and as per the study protocol.
Randomization	As this was a pilot clinical trial, it was not a randomized study.
Blinding	As this was a pilot clinical trial with a treatment intervention, blinding was not performed.

Reporting for specific materials, systems and methods

We require information from authors about some types of materials, experimental systems and methods used in many studies. Here, indicate whether each material, system or method listed is relevant to your study. If you are not sure if a list item applies to your research, read the appropriate section before selecting a response.

Materials & experimental systems

n/a	Involved in the study	
☒	☐	Antibodies
☒	☐	Eukaryotic cell lines
☒	☐	Palaeontology and archaeology
☒	☐	Animals and other organisms
☐	☒	Human research participants
☐	☒	Clinical data
☒	☐	Dual use research of concern

Methods

n/a	Involved in the study	
☒	☐	ChIP-seq
☒	☐	Flow cytometry
☒	☐	MRI-based neuroimaging

Human research participants

Policy information about studies involving human research participants

Population characteristics	Patients with stage I-III invasive breast cancer who had completed adjuvant or neo-adjuvant chemotherapy underwent unilateral bone marrow aspiration to screen for the presence of DTCs (Clinical Trials.gov Identifier: NCT00295867). Written informed consent was obtained from all patients. The study was conducted in accordance with the Declaration of Helsinki and Good Clinical Practice Guidelines. The institutional review board for The University of California San Francisco approved the study. Women over the age of 18 years with stage I-III breast cancer (confirmed histologically or cytologically) who had completed neo-adjuvant or adjuvant chemotherapy (if recommended), and had > 4 DTC/mL in the bone marrow (performed at least 3 weeks after completion of any adjuvant chemotherapy or at diagnosis in patients not receiving any adjuvant therapy or hormonal therapy alone or those who had surgery following neo-adjuvant therapy for breast cancer) were eligible. Patients were required to have adequate renal function (creatinine ≤ upper limit of normal), normal liver function (total bilirubin, alkaline phosphatase, and aspartate aminotransferase). Patients could receive concomitant hormonal therapy and/or radiation (if indicated). Exclusion criteria included history of allergy to bisphosphonates (not including acute phase reaction), renal insufficiency (defined by a serum creatinine > than upper limit of normal or a creatinine clearance < 50 mL/min due to any underlying cause), Karnofsky Performance Status < 90%, pregnancy, and any significant comorbid medical condition that could interfere with treatment. The use of other bone directed therapy during the study period was not allowed.

Recruitment	Patients were recruited through breast oncology clinics at the University of California San Francisco under the guidance of a treating physician.
Ethics oversight	Written informed consent was obtained from all patients. The study was conducted in accordance with the Declaration of Helsinki and Good Clinical Practice Guidelines. The institutional review board for The University of California San Francisco approved the study.

Note that full information on the approval of the study protocol must also be provided in the manuscript.

Clinical data

Policy information about clinical studies

All manuscripts should comply with the ICMJE guidelines for publication of clinical research and a completed CONSORT checklist must be included with all submissions.

Clinical trial registration	Clinical Trials.gov Identifier: NCT00295867
Study protocol	Clinical Trials.gov Identifier: NCT00295867
Data collection	Patients were recruited to this study starting in 2008, and data collection started then until the last subject came off study.
Outcomes	The primary endpoint was achieving a complete DTC response, defined as reduction of DTCs to less than 4MM/mL.<br>Additional exploratory endpoints included reduction in DTCs over time, recurrence, survival and toxicity. Time to recurrence and the Fisher’s Exact test were used to assess the impact of baseline parameters of DTCs and CTCs on risk of recurrence and death. The linear mixed effects model was used to determine the change in urinary n-telopeptide over time.
